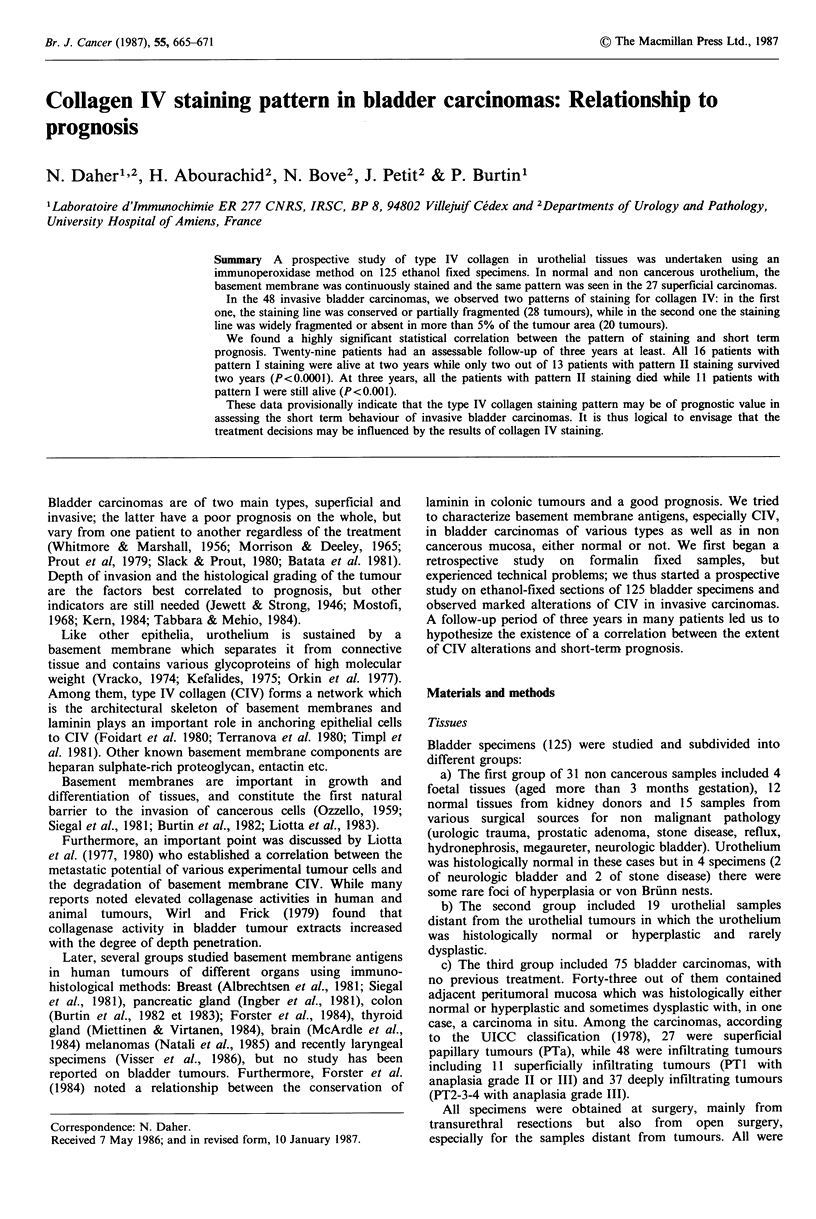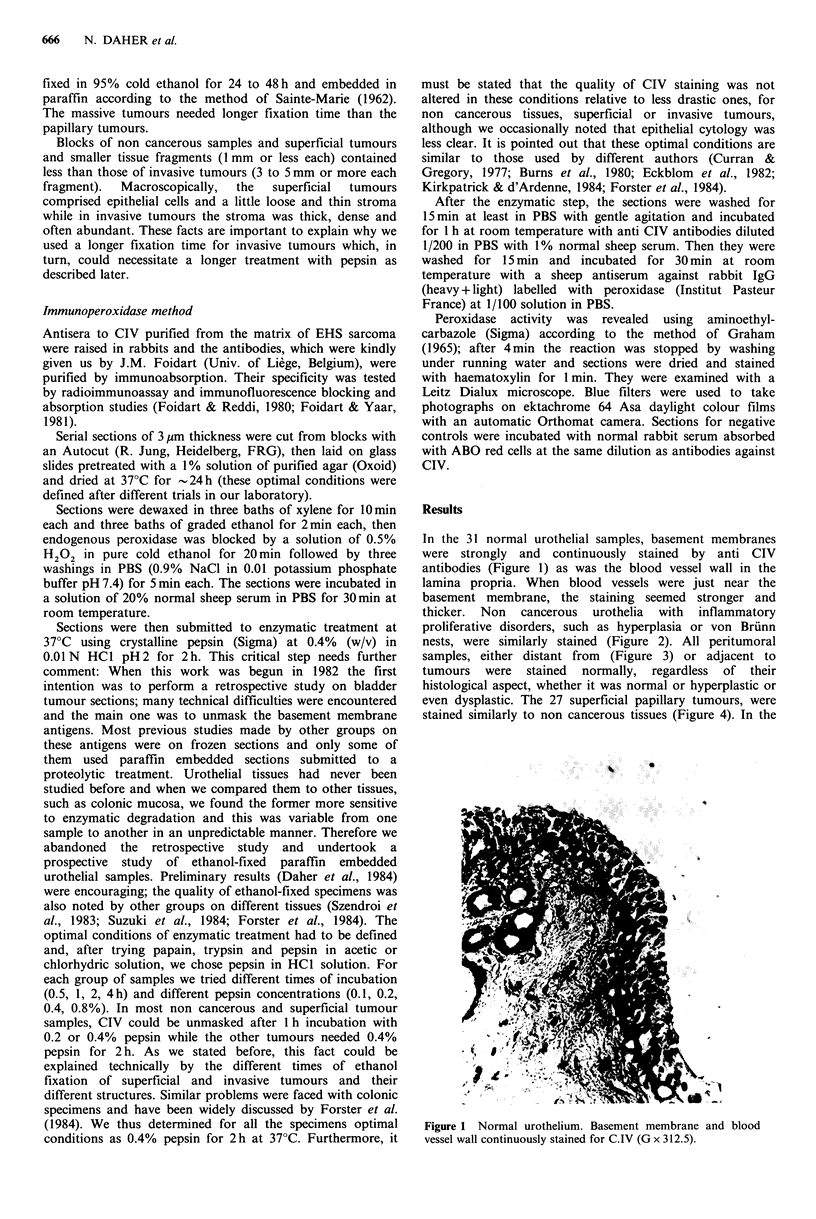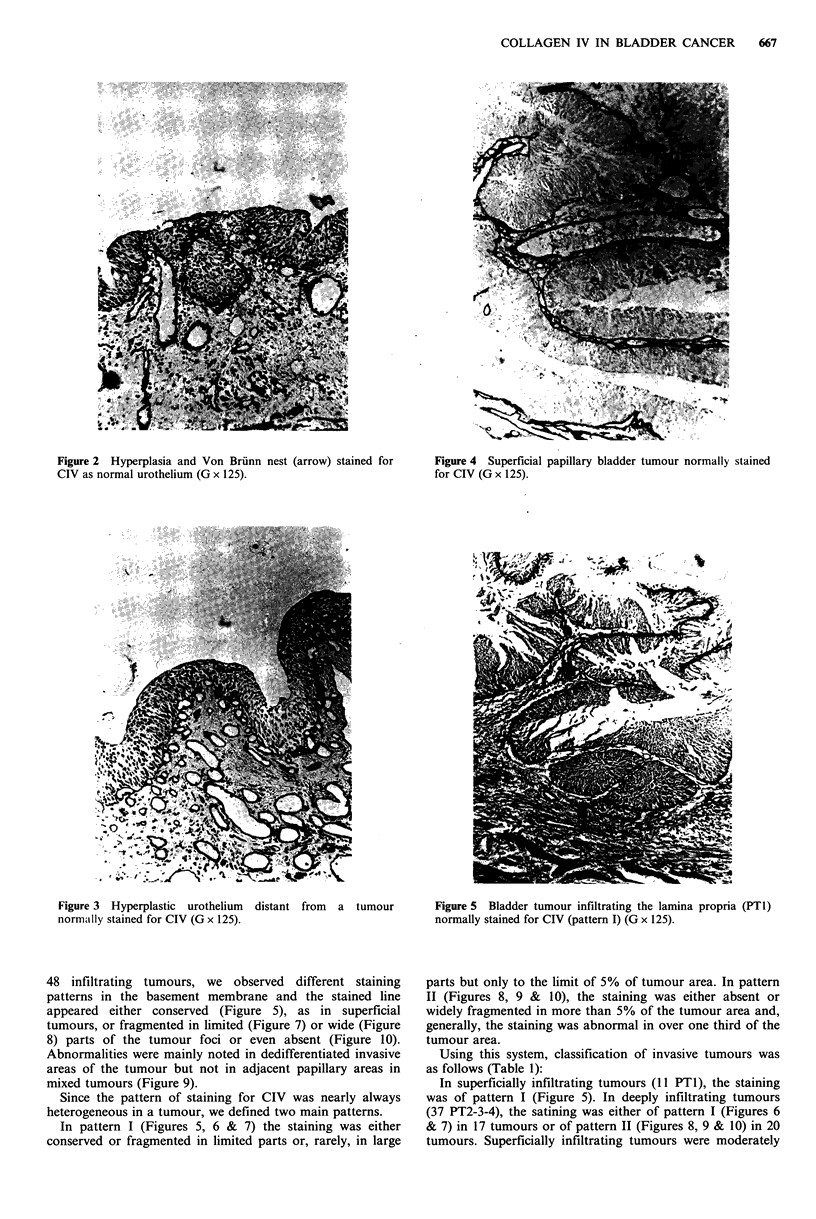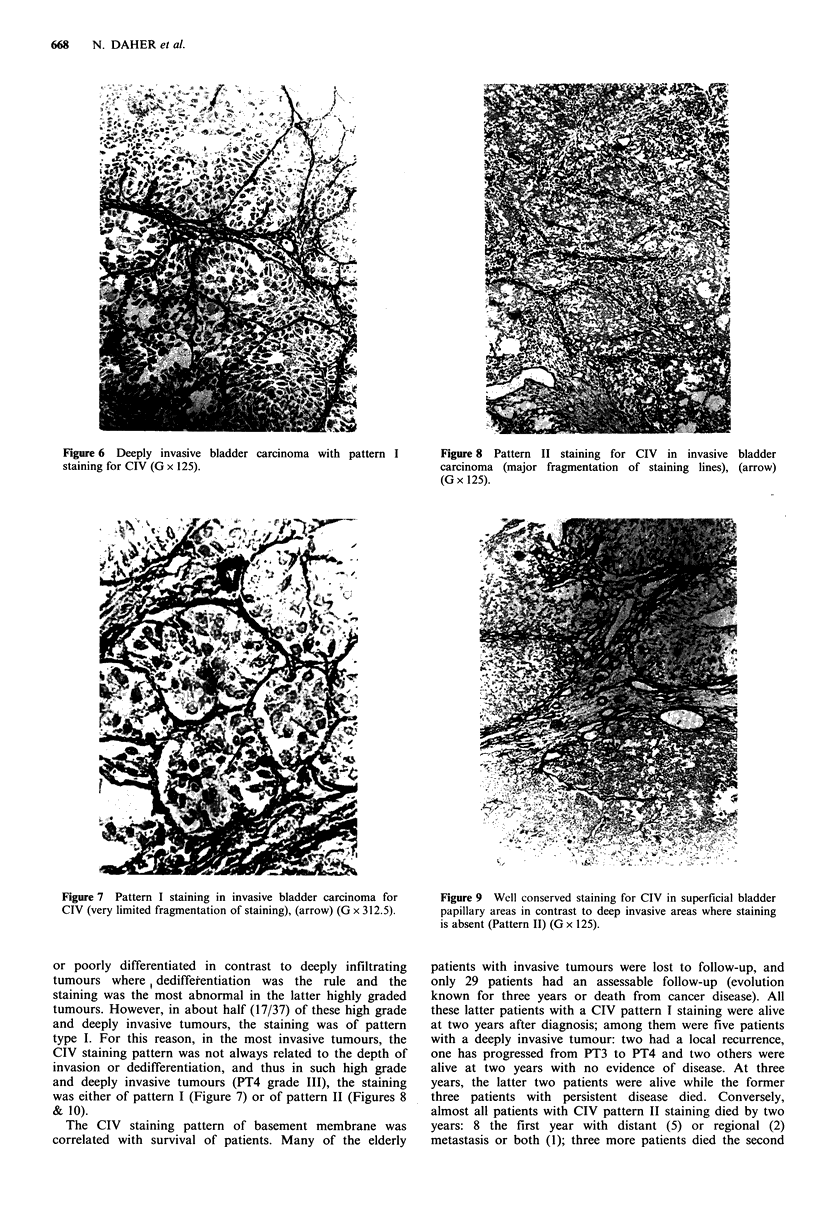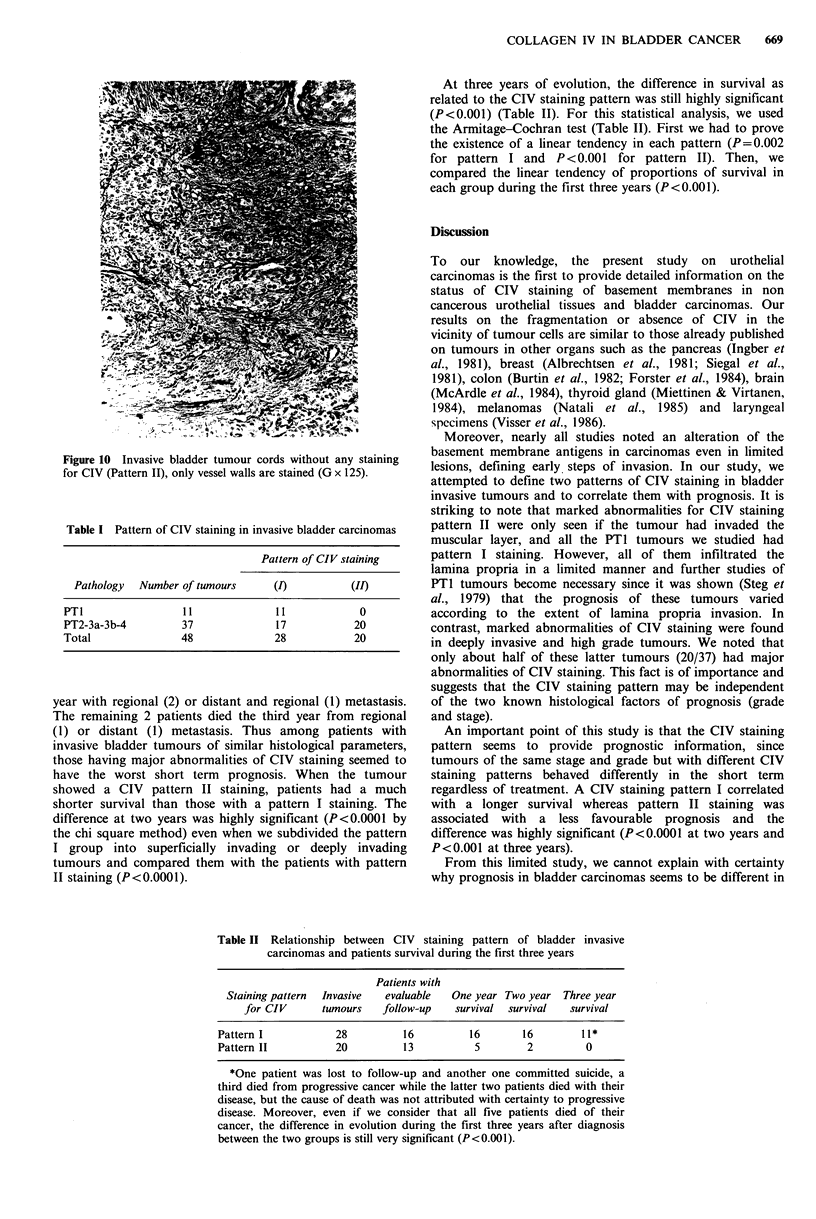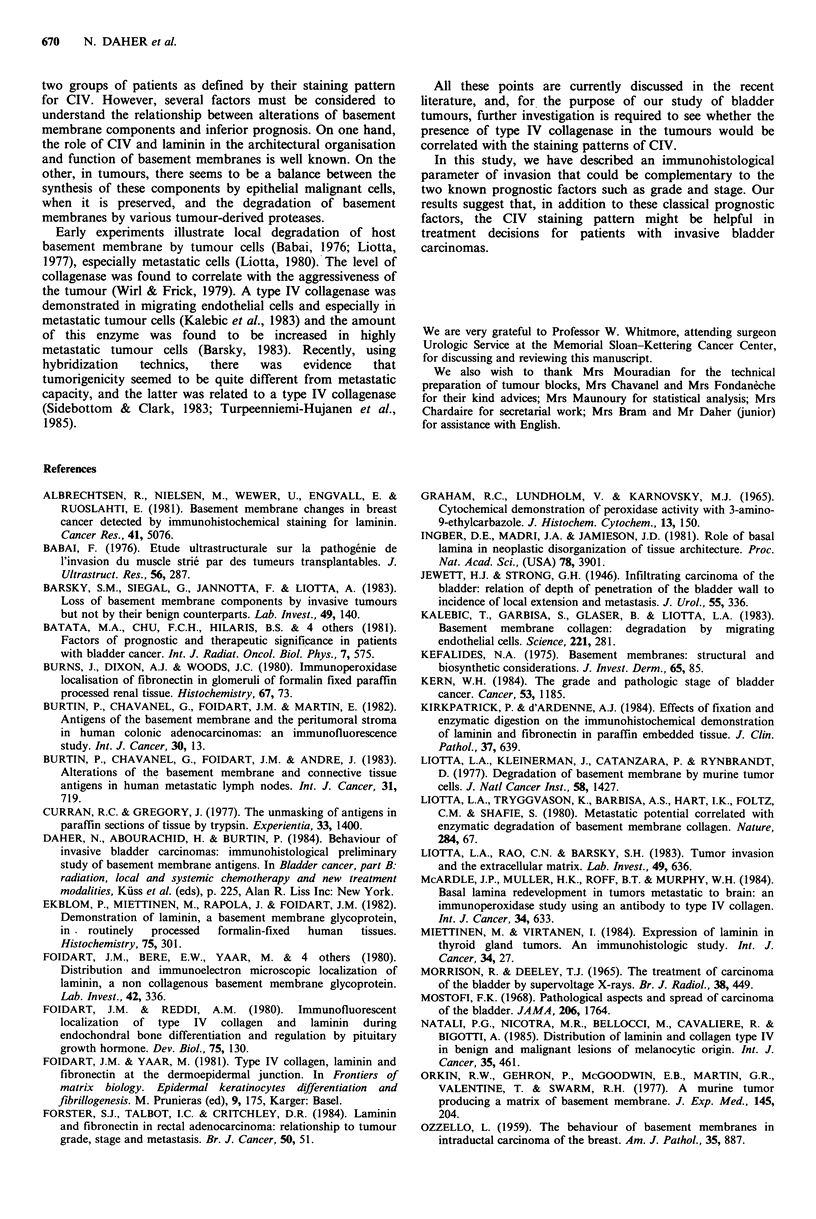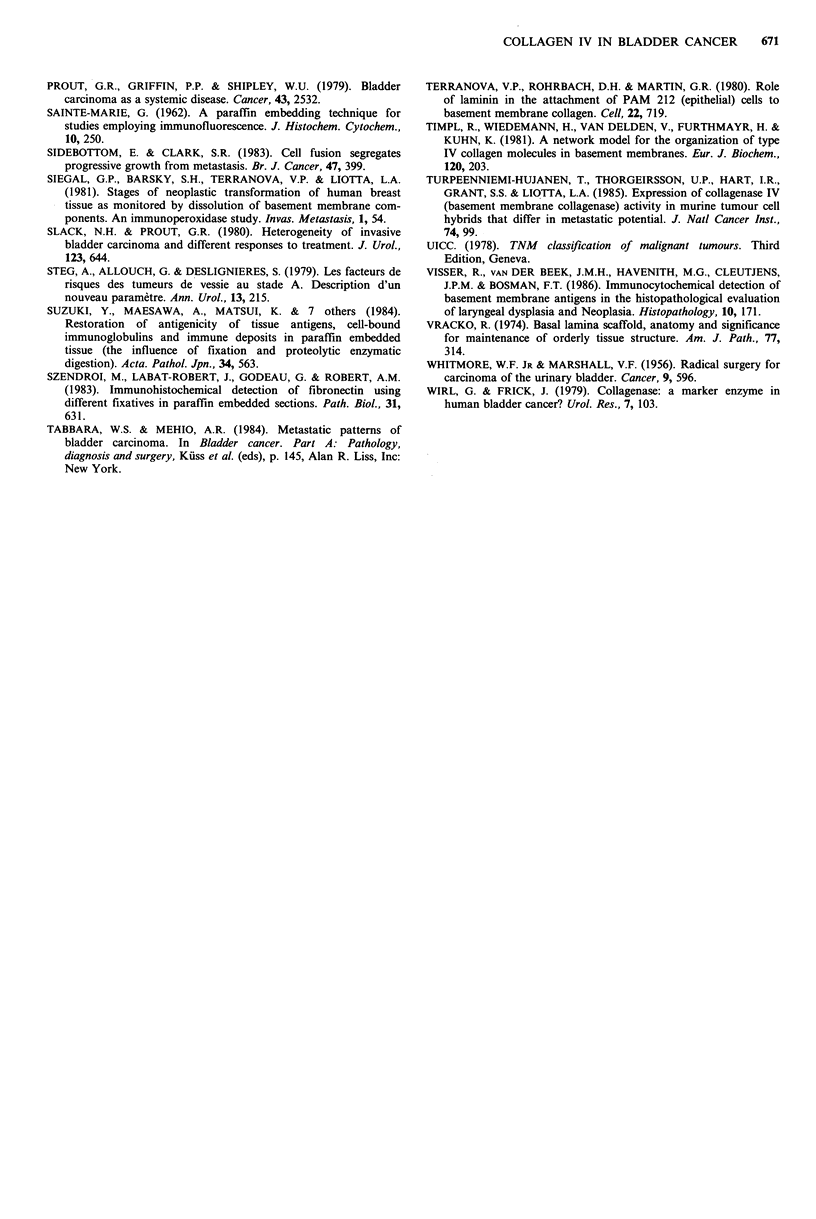# Collagen IV staining pattern in bladder carcinomas: relationship to prognosis.

**DOI:** 10.1038/bjc.1987.136

**Published:** 1987-06

**Authors:** N. Daher, H. Abourachid, N. Bove, J. Petit, P. Burtin

## Abstract

**Images:**


*Br. J. Cancer* (1987), **55**, 665–671 © The Macmillan Press Ltd., 1987

### Collagen IV staining pattern in bladder carcinomas: Relationship to prognosis

N. Daher[1,2], H. Abourachid[2], N. Bove[2], J. Petit[2] & P. Burtin[1]

[1]*Laboratoire d'Immunochimie ER 277 CNRS, IRSC, BP 8, 94802 Villejuif Cédex and* [2]*Departments of Urology and Pathology, University Hospital of Amiens, France*

**Summary** A prospective study of type IV collagen in urothelial tissues was undertaken using an immunoperoxidase method on 125 ethanol fixed specimens. In normal and non cancerous urothelium, the basement membrane was continuously stained and the same pattern was seen in the 27 superficial carcinomas.

In the 48 invasive bladder carcinomas, we observed two patterns of staining for collagen IV: in the first one, the staining line was conserved or partially fragmented (28 tumours), while in the second one the staining line was widely fragmented or absent in more than 5% of the tumour area (20 tumours).

We found a highly significant statistical correlation between the pattern of staining and short term prognosis. Twenty-nine patients had an assessable follow-up of three years at least. All 16 patients with pattern I staining were alive at two years while only two out of 13 patients with pattern II staining survived two years ($P<0.0001$). At three years, all the patients with pattern II staining died while 11 patients with pattern I were still alive ($P<0.001$).

These data provisionally indicate that the type IV collagen staining pattern may be of prognostic value in assessing the short term behaviour of invasive bladder carcinomas. It is thus logical to envisage that the treatment decisions may be influenced by the results of collagen IV staining.

Bladder carcinomas are of two main types, superficial and invasive; the latter have a poor prognosis on the whole, but vary from one patient to another regardless of the treatment (Whitmore & Marshall, 1956; Morrison & Deeley, 1965; Prout *et al*, 1979; Slack & Prout, 1980; Batata *et al.* 1981). Depth of invasion and the histological grading of the tumour are the factors best correlated to prognosis, but other indicators are still needed (Jewett & Strong, 1946; Mostofi, 1968; Kern, 1984; Tabbara & Mehio, 1984).

Like other epithelia, urothelium is sustained by a basement membrane which separates it from connective tissue and contains various glycoproteins of high molecular weight (Vracko, 1974; Kefalides, 1975; Orkin *et al.* 1977). Among them, type IV collagen (CIV) forms a network which is the architectural skeleton of basement membranes and laminin plays an important role in anchoring epithelial cells to CIV (Foidart *et al.* 1980; Terranova *et al.* 1980; Timpl *et al.* 1981). Other known basement membrane components are heparan sulphate-rich proteoglycan, entactin etc.

Basement membranes are important in growth and differentiation of tissues, and constitute the first natural barrier to the invasion of cancerous cells (Ozzello, 1959; Siegal *et al.*, 1981; Burtin *et al.*, 1982; Liotta *et al.*, 1983).

Furthermore, an important point was discussed by Liotta *et al.* (1977, 1980) who established a correlation between the metastatic potential of various experimental tumour cells and the degradation of basement membrane CIV. While many reports noted elevated collagenase activities in human and animal tumours, Wirl and Frick (1979) found that collagenase activity in bladder tumour extracts increased with the degree of depth penetration.

Later, several groups studied basement membrane antigens in human tumours of different organs using immunohistological methods: Breast (Albrechtsen *et al.*, 1981; Siegal *et al.*, 1981), pancreatic gland (Ingber *et al.*, 1981), colon (Burtin *et al.*, 1982 et 1983); Forster *et al.*, 1984), thyroid gland (Miettinen & Virtanen, 1984), brain (McArdle *et al.*, 1984) melanomas (Natali *et al.*, 1985) and recently laryngeal specimens (Visser *et al.*, 1986), but no study has been reported on bladder tumours. Furthermore, Forster *et al.* (1984) noted a relationship between the conservation of laminin in colonic tumours and a good prognosis. We tried to characterize basement membrane antigens, especially CIV, in bladder carcinomas of various types as well as in non cancerous mucosa, either normal or not. We first began a retrospective study on formalin fixed samples, but experienced technical problems; we thus started a prospective study on ethanol-fixed sections of 125 bladder specimens and observed marked alterations of CIV in invasive carcinomas. A follow-up period of three years in many patients led us to hypothesize the existence of a correlation between the extent of CIV alterations and short-term prognosis.

### Materials and methods

### *Tissues*

Bladder specimens (125) were studied and subdivided into different groups:

a) The first group of 31 non cancerous samples included 4 foetal tissues (aged more than 3 months gestation), 12 normal tissues from kidney donors and 15 samples from various surgical sources for non malignant pathology (urologic trauma, prostatic adenoma, stone disease, reflux, hydronephrosis, megaureter, neurologic bladder). Urothelium was histologically normal in these cases but in 4 specimens (2 of neurologic bladder and 2 of stone disease) there were some rare foci of hyperplasia or von Brünn nests.

b) The second group included 19 urothelial samples distant from the urothelial tumours in which the urothelium was histologically normal or hyperplastic and rarely dysplastic.

c) The third group included 75 bladder carcinomas, with no previous treatment. Forty-three out of them contained adjacent peritumoral mucosa which was histologically either normal or hyperplastic and sometimes dysplastic with, in one case, a carcinoma in situ. Among the carcinomas, according to the UICC classification (1978), 27 were superficial papillary tumours (PTa), while 48 were infiltrating tumours including 11 superficially infiltrating tumours (PT1 with anaplasia grade II or III) and 37 deeply infiltrating tumours (PT2-3-4 with anaplasia grade III).

All specimens were obtained at surgery, mainly from transurethral resections but also from open surgery, especially for the samples distant from tumours. All were

Correspondence: N. Daher.
Received 7 May 1986; and in revised form, 10 January 1987.

fixed in 95% cold ethanol for 24 to 48 h and embedded in paraffin according to the method of Sainte-Marie (1962). The massive tumours needed longer fixation time than the papillary tumours.

Blocks of non cancerous samples and superficial tumours and smaller tissue fragments (1 mm or less each) contained less than those of invasive tumours (3 to 5 mm or more each fragment). Macroscopically, the superficial tumours comprised epithelial cells and a little loose and thin stroma while in invasive tumours the stroma was thick, dense and often abundant. These facts are important to explain why we used a longer fixation time for invasive tumours which, in turn, could necessitate a longer treatment with pepsin as described later.

*Immunoperoxidase method*

Antisera to CIV purified from the matrix of EHS sarcoma were raised in rabbits and the antibodies, which were kindly given us by J.M. Foidart (Univ. of Liège, Belgium), were purified by immunoabsorption. Their specificity was tested by radioimmunoassay and immunofluorescence blocking and absorption studies (Foidart & Reddi, 1980; Foidart & Yaar, 1981).

Serial sections of 3 $\mu$m thickness were cut from blocks with an Autocut (R. Jung, Heidelberg, FRG), then laid on glass slides pretreated with a 1% solution of purified agar (Oxoid) and dried at 37°C for ~24 h (these optimal conditions were defined after different trials in our laboratory).

Sections were dewaxed in three baths of xylene for 10 min each and three baths of graded ethanol for 2 min each, then endogenous peroxidase was blocked by a solution of 0.5% $H_2O_2$ in pure cold ethanol for 20 min followed by three washings in PBS (0.9% NaCl in 0.01 potassium phosphate buffer pH 7.4) for 5 min each. The sections were incubated in a solution of 20% normal sheep serum in PBS for 30 min at room temperature.

Sections were then submitted to enzymatic treatment at 37°C using crystalline pepsin (Sigma) at 0.4% (w/v) in 0.01 N HCl pH 2 for 2 h. This critical step needs further comment: When this work was begun in 1982 the first intention was to perform a retrospective study on bladder tumour sections; many technical difficulties were encountered and the main one was to unmask the basement membrane antigens. Most previous studies made by other groups on these antigens were on frozen sections and only some of them used paraffin embedded sections submitted to a proteolytic treatment. Urothelial tissues had never been studied before and when we compared them to other tissues, such as colonic mucosa, we found the former more sensitive to enzymatic degradation and this was variable from one sample to another in an unpredictable manner. Therefore we abandoned the retrospective study and undertook a prospective study of ethanol-fixed paraffin embedded urothelial samples. Preliminary results (Daher et al., 1984) were encouraging; the quality of ethanol-fixed specimens was also noted by other groups on different tissues (Szendroi et al., 1983; Suzuki et al., 1984; Forster et al., 1984). The optimal conditions of enzymatic treatment had to be defined and, after trying papain, trypsin and pepsin in acetic or chlorhydric solution, we chose pepsin in HCl solution. For each group of samples we tried different times of incubation (0.5, 1, 2, 4 h) and different pepsin concentrations (0.1, 0.2, 0.4, 0.8%). In most non cancerous and superficial tumour samples, CIV could be unmasked after 1 h incubation with 0.2 or 0.4% pepsin while the other tumours needed 0.4% pepsin for 2 h. As we stated before, this fact could be explained technically by the different times of ethanol fixation of superficial and invasive tumours and their different structures. Similar problems were faced with colonic specimens and have been widely discussed by Forster et al. (1984). We thus determined for all the specimens optimal conditions as 0.4% pepsin for 2 h at 37°C. Furthermore, it must be stated that the quality of CIV staining was not altered in these conditions relative to less drastic ones, for non cancerous tissues, superficial or invasive tumours, although we occasionally noted that epithelial cytology was less clear. It is pointed out that these optimal conditions are similar to those used by different authors (Curran & Gregory, 1977; Burns et al., 1980; Eckblom et al., 1982; Kirkpatrick & d'Ardenne, 1984; Forster et al., 1984).

After the enzymatic step, the sections were washed for 15 min at least in PBS with gentle agitation and incubated for 1 h at room temperature with anti CIV antibodies diluted 1/200 in PBS with 1% normal sheep serum. Then they were washed for 15 min and incubated for 30 min at room temperature with a sheep antiserum against rabbit IgG (heavy + light) labelled with peroxidase (Institut Pasteur France) at 1/100 solution in PBS.

Peroxidase activity was revealed using aminoethylcarbazole (Sigma) according to the method of Graham (1965); after 4 min the reaction was stopped by washing under running water and sections were dried and stained with haematoxylin for 1 min. They were examined with a Leitz Dialux microscope. Blue filters were used to take photographs on ektachrome 64 Asa daylight colour films with an automatic Orthomat camera. Sections for negative controls were incubated with normal rabbit serum absorbed with ABO red cells at the same dilution as antibodies against CIV.

### Results

In the 31 normal urothelial samples, basement membranes were strongly and continuously stained by anti CIV antibodies (Figure 1) as was the blood vessel wall in the lamina propria. When blood vessels were just near the basement membrane, the staining seemed stronger and thicker. Non cancerous urothelia with inflammatory proliferative disorders, such as hyperplasia or von Brünn nests, were similarly stained (Figure 2). All peritumoral samples, either distant from (Figure 3) or adjacent to tumours were stained normally, regardless of their histological aspect, whether it was normal or hyperplastic or even dysplastic. The 27 superficial papillary tumours, were stained similarly to non cancerous tissues (Figure 4). In the

**Figure 1** Normal urothelium. Basement membrane and blood vessel wall continuously stained for C.IV (G × 312.5).

**Figure 2** Hyperplasia and Von Brünn nest (arrow) stained for CIV as normal urothelium (G x 125).

**Figure 4** Superficial papillary bladder tumour normally stained for CIV (G x 125).

**Figure 3** Hyperplastic urothelium distant from a tumour normally stained for CIV (G x 125).

**Figure 5** Bladder tumour infiltrating the lamina propria (PT1) normally stained for CIV (pattern I) (G x 125).

48 infiltrating tumours, we observed different staining patterns in the basement membrane and the stained line appeared either conserved (Figure 5), as in superficial tumours, or fragmented in limited (Figure 7) or wide (Figure 8) parts of the tumour foci or even absent (Figure 10). Abnormalities were mainly noted in dedifferentiated invasive areas of the tumour but not in adjacent papillary areas in mixed tumours (Figure 9).

Since the pattern of staining for CIV was nearly always heterogeneous in a tumour, we defined two main patterns.

In pattern I (Figures 5, 6 & 7) the staining was either conserved or fragmented in limited parts or, rarely, in large parts but only to the limit of 5% of tumour area. In pattern II (Figures 8, 9 & 10), the staining was either absent or widely fragmented in more than 5% of the tumour area and, generally, the staining was abnormal in over one third of the tumour area.

Using this system, classification of invasive tumours was as follows (Table 1):

In superficially infiltrating tumours (11 PT1), the staining was of pattern I (Figure 5). In deeply infiltrating tumours (37 PT2-3-4), the satining was either of pattern I (Figures 6 & 7) in 17 tumours or of pattern II (Figures 8, 9 & 10) in 20 tumours. Superficially infiltrating tumours were moderately

**Figure 6** Deeply invasive bladder carcinoma with pattern I staining for CIV (G x 125).

**Figure 8** Pattern II staining for CIV in invasive bladder carcinoma (major fragmentation of staining lines), (arrow) (G x 125).

**Figure 7** Pattern I staining in invasive bladder carcinoma for CIV (very limited fragmentation of staining), (arrow) (G x 312.5).

**Figure 9** Well conserved staining for CIV in superficial bladder papillary areas in contrast to deep invasive areas where staining is absent (Pattern II) (G x 125).

or poorly differentiated in contrast to deeply infiltrating tumours where dedifferentiation was the rule and the staining was the most abnormal in the latter highly graded tumours. However, in about half (17/37) of these high grade and deeply invasive tumours, the staining was of pattern type I. For this reason, in the most invasive tumours, the CIV staining pattern was not always related to the depth of invasion or dedifferentiation, and thus in such high grade and deeply invasive tumours (PT4 grade III), the staining was either of pattern I (Figure 7) or of pattern II (Figures 8 & 10).

The CIV staining pattern of basement membrane was correlated with survival of patients. Many of the elderly patients with invasive tumours were lost to follow-up, and only 29 patients had an assessable follow-up (evolution known for three years or death from cancer disease). All these latter patients with a CIV pattern I staining were alive at two years after diagnosis; among them were five patients with a deeply invasive tumour: two had a local recurrence, one has progressed from PT3 to PT4 and two others were alive at two years with no evidence of disease. At three years, the latter two patients were alive while the former three patients with persistent disease died. Conversely, almost all patients with CIV pattern II staining died by two years: 8 the first year with distant (5) or regional (2) metastasis or both (1); three more patients died the second

**Figure 10** Invasive bladder tumour cords without any staining for CIV (Pattern II), only vessel walls are stained (G x 125).

**Table I** Pattern of CIV staining in invasive bladder carcinomas

| *Pathology* | *Number of tumours* | *Pattern of CIV staining* (*I*) | (*II*) |
|---|---|---|---|
| PT1 | 11 | 11 | 0 |
| PT2-3a-3b-4 | 37 | 17 | 20 |
| Total | 48 | 28 | 20 |

year with regional (2) or distant and regional (1) metastasis. The remaining 2 patients died the third year from regional (1) or distant (1) metastasis. Thus among patients with invasive bladder tumours of similar histological parameters, those having major abnormalities of CIV staining seemed to have the worst short term prognosis. When the tumour showed a CIV pattern II staining, patients had a much shorter survival than those with a pattern I staining. The difference at two years was highly significant ($P<0.0001$ by the chi square method) even when we subdivided the pattern I group into superficially invading or deeply invading tumours and compared them with the patients with pattern II staining ($P<0.0001$).

At three years of evolution, the difference in survival as related to the CIV staining pattern was still highly significant ($P<0.001$) (Table II). For this statistical analysis, we used the Armitage–Cochran test (Table II). First we had to prove the existence of a linear tendency in each pattern ($P=0.002$ for pattern I and $P<0.001$ for pattern II). Then, we compared the linear tendency of proportions of survival in each group during the first three years ($P<0.001$).

### Discussion

To our knowledge, the present study on urothelial carcinomas is the first to provide detailed information on the status of CIV staining of basement membranes in non cancerous urothelial tissues and bladder carcinomas. Our results on the fragmentation or absence of CIV in the vicinity of tumour cells are similar to those already published on tumours in other organs such as the pancreas (Ingber *et al.*, 1981), breast (Albrechtsen *et al.*, 1981; Siegal *et al.*, 1981), colon (Burtin *et al.*, 1982; Forster *et al.*, 1984), brain (McArdle *et al.*, 1984), thyroid gland (Miettinen & Virtanen, 1984), melanomas (Natali *et al.*, 1985) and laryngeal specimens (Visser *et al.*, 1986).

Moreover, nearly all studies noted an alteration of the basement membrane antigens in carcinomas even in limited lesions, defining early steps of invasion. In our study, we attempted to define two patterns of CIV staining in bladder invasive tumours and to correlate them with prognosis. It is striking to note that marked abnormalities for CIV staining pattern II were only seen if the tumour had invaded the muscular layer, and all the PT1 tumours we studied had pattern I staining. However, all of them infiltrated the lamina propria in a limited manner and further studies of PT1 tumours become necessary since it was shown (Steg *et al.*, 1979) that the prognosis of these tumours varied according to the extent of lamina propria invasion. In contrast, marked abnormalities of CIV staining were found in deeply invasive and high grade tumours. We noted that only about half of these latter tumours (20/37) had major abnormalities of CIV staining. This fact is of importance and suggests that the CIV staining pattern may be independent of the two known histological factors of prognosis (grade and stage).

An important point of this study is that the CIV staining pattern seems to provide prognostic information, since tumours of the same stage and grade but with different CIV staining patterns behaved differently in the short term regardless of treatment. A CIV staining pattern I correlated with a longer survival whereas pattern II staining was associated with a less favourable prognosis and the difference was highly significant ($P<0.0001$ at two years and $P<0.001$ at three years).

From this limited study, we cannot explain with certainty why prognosis in bladder carcinomas seems to be different in

**Table II** Relationship between CIV staining pattern of bladder invasive carcinomas and patients survival during the first three years

| *Staining pattern for CIV* | *Invasive tumours* | *Patients with evaluable follow-up* | *One year survival* | *Two year survival* | *Three year survival* |
|---|---|---|---|---|---|
| Pattern I | 28 | 16 | 16 | 16 | 11* |
| Pattern II | 20 | 13 | 5 | 2 | 0 |

*One patient was lost to follow-up and another one committed suicide, a third died from progressive cancer while the latter two patients died with their disease, but the cause of death was not attributed with certainty to progressive disease. Moreover, even if we consider that all five patients died of their cancer, the difference in evolution during the first three years after diagnosis between the two groups is still very significant ($P<0.001$).

two groups of patients as defined by their staining pattern for CIV. However, several factors must be considered to understand the relationship between alterations of basement membrane components and inferior prognosis. On one hand, the role of CIV and laminin in the architectural organisation and function of basement membranes is well known. On the other, in tumours, there seems to be a balance between the synthesis of these components by epithelial malignant cells, when it is preserved, and the degradation of basement membranes by various tumour-derived proteases.

Early experiments illustrate local degradation of host basement membrane by tumour cells (Babai, 1976; Liotta, 1977), especially metastatic cells (Liotta, 1980). The level of collagenase was found to correlate with the aggressiveness of the tumour (Wirl & Frick, 1979). A type IV collagenase was demonstrated in migrating endothelial cells and especially in metastatic tumour cells (Kalebic *et al.*, 1983) and the amount of this enzyme was found to be increased in highly metastatic tumour cells (Barsky, 1983). Recently, using hybridization technics, there was evidence that tumorigenicity seemed to be quite different from metastatic capacity, and the latter was related to a type IV collagenase (Sidebottom & Clark, 1983; Turpeenniemi-Hujanen *et al.*, 1985).

All these points are currently discussed in the recent literature, and, for the purpose of our study of bladder tumours, further investigation is required to see whether the presence of type IV collagenase in the tumours would be correlated with the staining patterns of CIV.

In this study, we have described an immunohistological parameter of invasion that could be complementary to the two known prognostic factors such as grade and stage. Our results suggest that, in addition to these classical prognostic factors, the CIV staining pattern might be helpful in treatment decisions for patients with invasive bladder carcinomas.

We are very grateful to Professor W. Whitmore, attending surgeon Urologic Service at the Memorial Sloan–Kettering Cancer Center, for discussing and reviewing this manuscript.

We also wish to thank Mrs Mouradian for the technical preparation of tumour blocks, Mrs Chavanel and Mrs Fondanèche for their kind advices; Mrs Maunoury for statistical analysis; Mrs Chardaire for secretarial work; Mrs Bram and Mr Daher (junior) for assistance with English.

### References

ALBRECHTSEN, R., NIELSEN, M., WEWER, U., ENGVALL, E. & RUOSLAHTI, E. (1981). Basement membrane changes in breast cancer detected by immunohistochemical staining for laminin. *Cancer Res.*, **41**, 5076.

BABAI, F. (1976). Etude ultrastructurale sur la pathogénie de l'invasion du muscle strié par des tumeurs transplantables. *J. Ultrastruct. Res.*, **56**, 287.

BARSKY, S.M., SIEGAL, G., JANNOTTA, F. & LIOTTA, A. (1983). Loss of basement membrane components by invasive tumours but not by their benign counterparts. *Lab. Invest.*, **49**, 140.

BATATA, M.A., CHU, F.C.H., HILARIS, B.S. & 4 others (1981). Factors of prognostic and therapeutic significance in patients with bladder cancer. *Int. J. Radiat. Oncol. Biol. Phys.*, **7**, 575.

BURNS, J., DIXON, A.J. & WOODS, J.C. (1980). Immunoperoxidase localisation of fibronectin in glomeruli of formalin fixed paraffin processed renal tissue. *Histochemistry*, **67**, 73.

BURTIN, P., CHAVANEL, G., FOIDART, J.M. & MARTIN, E. (1982). Antigens of the basement membrane and the peritumoral stroma in human colonic adenocarcinomas: an immunofluorescence study. *Int. J. Cancer*, **30**, 13.

BURTIN, P., CHAVANEL, G., FOIDART, J.M. & ANDRE, J. (1983). Alterations of the basement membrane and connective tissue antigens in human metastatic lymph nodes. *Int. J. Cancer*, **31**, 719.

CURRAN, R.C. & GREGORY, J. (1977). The unmasking of antigens in paraffin sections of tissue by trypsin. *Experientia*, **33**, 1400.

DAHER, N., ABOURACHID, H. & BURTIN, P. (1984). Behaviour of invasive bladder carcinomas: immunohistological preliminary study of basement membrane antigens. In *Bladder cancer, part B: radiation, local and systemic chemotherapy and new treatment modalities*, Küss *et al.* (eds), p. 225, Alan R. Liss Inc: New York.

EKBLOM, P., MIETTINEN, M., RAPOLA, J. & FOIDART, J.M. (1982). Demonstration of laminin, a basement membrane glycoprotein, in routinely processed formalin-fixed human tissues. *Histochemistry*, **75**, 301.

FOIDART, J.M., BERE, E.W., YAAR, M. & 4 others (1980). Distribution and immunoelectron microscopic localization of laminin, a non collagenous basement membrane glycoprotein. *Lab. Invest.*, **42**, 336.

FOIDART, J.M. & REDDI, A.M. (1980). Immunofluorescent localization of type IV collagen and laminin during endochondral bone differentiation and regulation by pituitary growth hormone. *Dev. Biol.*, **75**, 130.

FOIDART, J.M. & YAAR, M. (1981). Type IV collagen, laminin and fibronectin at the dermoepidermal junction. In *Frontiers of matrix biology. Epidermal keratinocytes differentiation and fibrillogenesis.* M. Prunieras (ed), **9**, 175, Karger: Basel.

FORSTER, S.J., TALBOT, I.C. & CRITCHLEY, D.R. (1984). Laminin and fibronectin in rectal adenocarcinoma: relationship to tumour grade, stage and metastasis. *Br. J. Cancer*, **50**, 51.

GRAHAM, R.C., LUNDHOLM, V. & KARNOVSKY, M.J. (1965). Cytochemical demonstration of peroxidase activity with 3-amino-9-ethylcarbazole. *J. Histochem. Cytochem.*, **13**, 150.

INGBER, D.E., MADRI, J.A. & JAMIESON, J.D. (1981). Role of basal lamina in neoplastic disorganization of tissue architecture. *Proc. Nat. Acad. Sci.*, (USA) **78**, 3901.

JEWETT, H.J. & STRONG, G.H. (1946). Infiltrating carcinoma of the bladder: relation of depth of penetration of the bladder wall to incidence of local extension and metastasis. *J. Urol.*, **55**, 336.

KALEBIC, T., GARBISA, S., GLASER, B. & LIOTTA, L.A. (1983). Basement membrane collagen: degradation by migrating endothelial cells. *Science*, **221**, 281.

KEFALIDES, N.A. (1975). Basement membranes: structural and biosynthetic considerations. *J. Invest. Derm.*, **65**, 85.

KERN, W.H. (1984). The grade and pathologic stage of bladder cancer. *Cancer*, **53**, 1185.

KIRKPATRICK, P. & d'ARDENNE, A.J. (1984). Effects of fixation and enzymatic digestion on the immunohistochemical demonstration of laminin and fibronectin in paraffin embedded tissue. *J. Clin. Pathol.*, **37**, 639.

LIOTTA, L.A., KLEINERMAN, J., CATANZARA, P. & RYNBRANDT, D. (1977). Degradation of basement membrane by murine tumor cells. *J. Natl Cancer Inst.*, **58**, 1427.

LIOTTA, L.A., TRYGGVASON, K., BARBISA, A.S., HART, I.K., FOLTZ, C.M. & SHAFIE, S. (1980). Metastatic potential correlated with enzymatic degradation of basement membrane collagen. *Nature*, **284**, 67.

LIOTTA, L.A., RAO, C.N. & BARSKY, S.H. (1983). Tumor invasion and the extracellular matrix. *Lab. Invest.*, **49**, 636.

McARDLE, J.P., MULLER, H.K., ROFF, B.T. & MURPHY, W.H. (1984). Basal lamina redevelopment in tumors metastatic to brain: an immunoperoxidase study using an antibody to type IV collagen. *Int. J. Cancer*, **34**, 633.

MIETTINEN, M. & VIRTANEN, I. (1984). Expression of laminin in thyroid gland tumors. An immunohistologic study. *Int. J. Cancer*, **34**, 27.

MORRISON, R. & DEELEY, T.J. (1965). The treatment of carcinoma of the bladder by supervoltage X-rays. *Br. J. Radiol.*, **38**, 449.

MOSTOFI, F.K. (1968). Pathological aspects and spread of carcinoma of the bladder. *JAMA*, **206**, 1764.

NATALI, P.G., NICOTRA, M.R., BELLOCCI, M., CAVALIERE, R. & BIGOTTI, A. (1985). Distribution of laminin and collagen type IV in benign and malignant lesions of melanocytic origin. *Int. J. Cancer*, **35**, 461.

ORKIN, R.W., GEHRON, P., McGOODWIN, E.B., MARTIN, G.R., VALENTINE, T. & SWARM, R.H. (1977). A murine tumor producing a matrix of basement membrane. *J. Exp. Med.*, **145**, 204.

OZZELLO, L. (1959). The behaviour of basement membranes in intraductal carcinoma of the breast. *Am. J. Pathol.*, **35**, 887.

PROUT, G.R., GRIFFIN, P.P. & SHIPLEY, W.U. (1979). Bladder carcinoma as a systemic disease. *Cancer*, **43**, 2532.

SAINTE-MARIE, G. (1962). A paraffin embedding technique for studies employing immunofluorescence. *J. Histochem. Cytochem.*, **10**, 250.

SIDEBOTTOM, E. & CLARK, S.R. (1983). Cell fusion segregates progressive growth from metastasis. *Br. J. Cancer*, **47**, 399.

SIEGAL, G.P., BARSKY, S.H., TERRANOVA, V.P. & LIOTTA, L.A. (1981). Stages of neoplastic transformation of human breast tissue as monitored by dissolution of basement membrane components. An immunoperoxidase study. *Invas. Metastasis*, **1**, 54.

SLACK, N.H. & PROUT, G.R. (1980). Heterogeneity of invasive bladder carcinoma and different responses to treatment. *J. Urol.*, **123**, 644.

STEG, A., ALLOUCH, G. & DESLIGNIERES, S. (1979). Les facteurs de risques des tumeurs de vessie au stade A. Description d'un nouveau paramètre. *Ann. Urol.*, **13**, 215.

SUZUKI, Y., MAESAWA, A., MATSUI, K. & 7 others (1984). Restoration of antigenicity of tissue antigens, cell-bound immunoglobulins and immune deposits in paraffin embedded tissue (the influence of fixation and proteolytic enzymatic digestion). *Acta. Pathol. Jpn.*, **34**, 563.

SZENDROI, M., LABAT-ROBERT, J., GODEAU, G. & ROBERT, A.M. (1983). Immunohistochemical detection of fibronectin using different fixatives in paraffin embedded sections. *Path. Biol.*, **31**, 631.

TABBARA, W.S. & MEHIO, A.R. (1984). Metastatic patterns of bladder carcinoma. In *Bladder cancer. Part A: Pathology, diagnosis and surgery*, Küss *et al.* (eds), p. 145, Alan R. Liss, Inc: New York.

TERRANOVA, V.P., ROHRBACH, D.H. & MARTIN, G.R. (1980). Role of laminin in the attachment of PAM 212 (epithelial) cells to basement membrane collagen. *Cell*, **22**, 719.

TIMPL, R., WIEDEMANN, H., VAN DELDEN, V., FURTHMAYR, H. & KUHN, K. (1981). A network model for the organization of type IV collagen molecules in basement membranes. *Eur. J. Biochem.*, **120**, 203.

TURPEENNIEMI-HUJANEN, T., THORGEIRSSON, U.P., HART, I.R., GRANT, S.S. & LIOTTA, L.A. (1985). Expression of collagenase IV (basement membrane collagenase) activity in murine tumour cell hybrids that differ in metastatic potential. *J. Natl Cancer Inst.*, **74**, 99.

UICC. (1978). *TNM classification of malignant tumours*. Third Edition, Geneva.

VISSER, R., VAN DER BEEK, J.M.H., HAVENITH, M.G., CLEUTJENS, J.P.M. & BOSMAN, F.T. (1986). Immunocytochemical detection of basement membrane antigens in the histopathological evaluation of laryngeal dysplasia and Neoplasia. *Histopathology*, **10**, 171.

VRACKO, R. (1974). Basal lamina scaffold, anatomy and significance for maintenance of orderly tissue structure. *Am. J. Path.*, **77**, 314.

WHITMORE, W.F. JR & MARSHALL, V.F. (1956). Radical surgery for carcinoma of the urinary bladder. *Cancer*, **9**, 596.

WIRL, G. & FRICK, J. (1979). Collagenase: a marker enzyme in human bladder cancer? *Urol. Res.*, **7**, 103.